# Highlight selection of radiochemistry and radiopharmacy developments by editorial board

**DOI:** 10.1186/s41181-023-00218-y

**Published:** 2023-10-27

**Authors:** Jean DaSilva, Clemens Decristoforo, Robert H. Mach, Guy Bormans, Giuseppe Carlucci, Mohammed Al-Qahtani, Adriano Duatti, Antony D. Gee, Wiktor Szymanski, Sietske Rubow, Jeroen Hendrikx, Xing Yang, Hongmei Jia, Junbo Zhang, Peter Caravan, Hua Yang, Jan Rijn Zeevaart, Miguel Avila Rodriquez, Ralph Santos Oliveira, Marcela Zubillaga, Tamer Sakr, Sarah Spreckelmeyer

**Affiliations:** 1 University of Montreal, Montreal, Canada; 2 Medical University Innsbruck, Innsbruck, Austria; 3 University of Pennsylvania, Philadelphia, USA; 4 University of Leuven, Leuven, Belgium; 5 UCLA, Los Angeles, USA; 6 King Faisal Specialist Hospital and Research Center, Riad, Saudi Arabia; 7 University of Ferrara, Ferrara, Italy; 8 King’s College, London, UK; 9 University of Groningen, Groningen, The Netherlands; 10 Stellenbosch University, Stellenbosch, South Africa; 11 NKI, Amsterdam, The Netherlands; 12 Peking University First Hospital, Beijing, China; 13 Beijing Normal University, Beijing, China; 14 Massuchusetts General Hospital, Harvard University, Boston, USA; 15 TRIUMF, Vancouver, Canada; 16 Necsa, Pretoria, South Africa; 17 Universidad Nacional Autonoma de Mexico (UNAM), Mexico-City, Mexico; 18 Brazilian Association of Radiopharmacy Brazil, Brazilian Nuclear Energy Commission - Nuclear Engineering Institute, State University of Rio de Janeiro, Rio de Janeiro, Brazil; 19 University of Buenos Aires, Buenos Aires, Argentina; 20 Egyptian Atomic Energy Authority, Cairo, Egypt; 21 Department of Nuclear Medicine, Charité - Universitätsmedizin Berlin, Corporate Member of Freie Universität Berlin, Humboldt-Universität Zu Berlin, and Berlin Institute of Health, Augustenburger Platz 1, 13353 Berlin, Germany

**Keywords:** Highlight articles, Radiochemistry, Radiopharmacy, Radiopharmaceutical sciences, Nuclear medicine, Trends in radiopharmaceutical sciences

## Abstract

**Background:**

The Editorial Board of EJNMMI Radiopharmacy and Chemistry releases a biannual highlight commentary to update the readership on trends in the field of radiopharmaceutical development.

**Main body:**

This selection of highlights provides commentary on 21 different topics selected by each coauthoring Editorial Board member addressing a variety of aspects ranging from novel radiochemistry to first-in-human application of novel radiopharmaceuticals.

**Conclusion:**

Trends in radiochemistry and radiopharmacy are highlighted. Hot topics cover the entire scope of EJNMMI Radiopharmacy and Chemistry, demonstrating the progress in the research field in many aspects.

## Background

Each individual coauthoring member of the Editorial Board has selected to highlight an article that has appeared in the radiochemistry, radiopharmacy and imaging agent literature during the period January–July 2023. The aim of this collaborative initiative is to create a biyearly overview for the readers summarizing the latest trends and hot topics in the field.

## Chemoenzymatic synthesis of disaccharide-based PET tracers for bacterial infection imaging

*By Wiktor Szymanski*

The synthesis of polysaccharides is notoriously difficult, due to the challenges related to regio- and stereo-selectivity, which often require laborious protection/deprotection strategies. This is especially troublesome in the synthesis of glycan-based positron emission tomography (PET) tracers bearing short-lived isotopes, where the radiolabelling should be performed ideally as the final step, and any subsequent conversion should be avoided.

Realizing the efficiency and selectivity of chemoenzymatic approaches, the group of Wilson employed a series of disaccharide phosphorylases for the conversion of [^18^F]fluoro-deoxyglucose ([^18^F]FDG), the most commonly used clinical PET tracer, into disaccharides (Sorlin et al. 2023). With this biocatalytic approach, the authors have synthesized ^18^F-labelled disaccharides with decay-corrected radiochemical yields up to 96%.

**Fig. 1:**
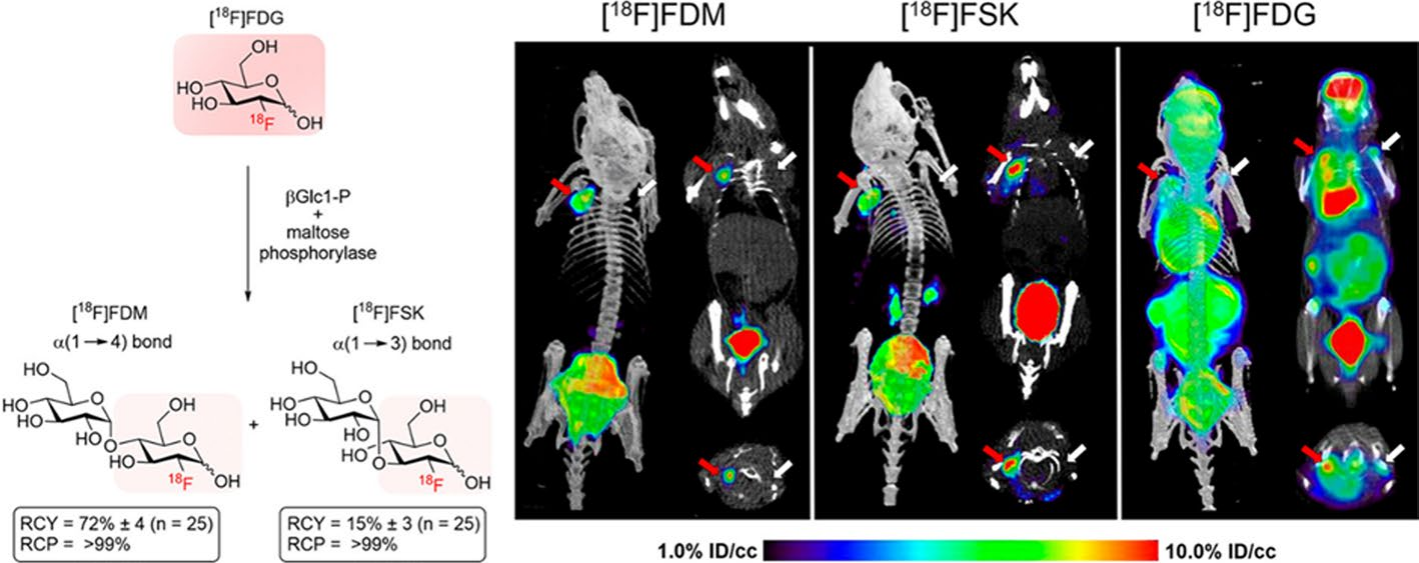
Chemoenzymatic, one step synthesis of [^18^F]FDM and [^18^F]FSK from [^18^F]FDG, and their selective uptake in an in vivo model of MRSA myositis (red arrow: site of inoculation with live bacteria; white arrow: site of inoculation with heat-killed bacteria). With permission from J Am Chem Soc 2023;145:17632–17642 (CC BY 4.0 License)

The most interesting products: 2-deoxy-[^18^F]-fluoro-maltose ([^18^F]FDM) and 2-deoxy-2-[^18^F]-fluoro-sakebiose ([^18^F]FSK) were then tested (see Fig. 1) in vitro and in vivo as selective tracers for the PET imaging of bacterial infections. The compounds showed accumulation in preclinical infection models, enabling the sensitive detection of *S. aureus*, including methicillin-resistant (MRSA) strains.

## Has ^225^Ac-labeled DOTA-JR11 the ability to improve the clinical outcome compared to ^177^Lu-DOTA-JR11?

*By Sarah Spreckelmeyer*

Alpha- and beta emitters are characterized by their well-known physical- and radiobiological features. The use of ^225^Ac-labelled radioconjugates like [^225^Ac]Ac-DOTA-TOC/TATE or [^225^Ac]Ac-PSMA is becoming clinically established, although big question marks regarding the assessment of radiochemical purity arise (Kelly et al. 2021; Kleynhans and Duatti 2022). ^177^Lu-mediated peptide receptor radionuclide therapies (PRRTs) of neuroendocrine tumors (NETs) fail sometimes due to resistances or disease recurrence. Alpha emitters such as ^225^Ac could offer an alternative approach, as highlighted in several studies (Yadav et al. 2022; Satapathy et al. 2021). Recently, Zimmermann prognosed an ^225^Ac production capacity by 2032 above 25 TBq, which might be sufficient for up to 2 million patients a year (Zimmermann 2023).

In a recent study (Handula et al. 2023), the somatostatin antagonist JR11 was radiolabeled with ^225^Ac and ^177^Lu and their in vitro and in vivo data were compared. As a result, the ^177^Lu analogue was more stable in mouse serum and PBS. The biodistribution profile of both compounds is similar, but the ^225^Ac-analogue showed a higher uptake in kidneys and bone marrow and the authors concluded that the JR11 analogue needs further optimization for reducing accumulation in off-target tissues. Additionally, they also highlighted, that the kidney uptake can be reduced by different strategies like the administration of an amino acid cocktail before application of the PRRT.

To conclude, [^225^Ac]Ac-DOTA-JR11 and other ^225^Ac-bioconcugates are expected to get more and more attention in the future. ^225^Ac-Labeled bioconjugates will likely eventually be used routinely to improve the clinical outcomes of patients.

## Radiochemistry and radiopharmacy in the era of artificial intelligence: glance to the future

*By Ralph Santos-Oliveira*

The use of artificial intelligence (AI) in the nuclear medicine field has emerged as a feasible possibility (Currie 2023). Despite the more routine use of AI in diagnosis and therapeutic planning (mostly dosimetric aspects), a new and potential use of AI is being applied in the strategic development of radiopharmaceuticals, either by planning the production of the radioisotope, or its application in complex chemical synthesis (radiochemistry) or in the pharmaceutical aspects of the formulation (radiopharmaceuticals itself) (Bradshaw et al. 2022). In this new era, the use of AI can be used for: (i) automated radiopharmaceutical production optimizing and automating the production of radiopharmaceuticals, turning the production processes streamlined, leading to increased efficiency and reduced human error; (ii) quality control of the radiopharmaceuticals used in the daily routine by processing images from several patients from various imaging techniques (e.g., PET, SPECT) to ensure that radiopharmaceuticals are produced within the required specifications and are safe for patient use; (iii) dose personalization by processing patient data to determine the optimal dosage of radiopharmaceuticals for individual patients, reducing the amount of radiation used, and consequently the radiation exposure in radiopharmaceutical cycle; (iv) radiochemistry synthesis can be performed more precisely, with the use of machine learning that will analyze big data generated during complex radiochemistry experiments in several different places, identifying patterns, correlations and trends, accelerating the development of new radiopharmaceuticals; (v) drug discovery and design by analyzing molecular structures, predicting binding affinities, and simulating interactions between potential compounds and biological targets. Besides all these applications the use of AI in the field of radiopharmacy can also include: (i) transportation control by the prediction of the decay and stability of the radiopharmaceuticals, suggesting the best route and/or transport to be used; (ii) workflow in industries and research laboratories and (iii) regulatory compliance, by the automated verification of documentation, to check if they are strictly adherent to the regulatory requirements.

Although new to the vast majority of us, AI is at our door, knocking to get in. It is up to us to learn to deal with this new reality and allow its use in the most correct, effective and ethical way possible.

## Initial evaluation of ^68^Ga-radiolabelled nanodiamonds

*By Mohammed Al-Qahtani*

A notable highlight from our field was performance of a full assessment on what can be called multi-modal targeting probes through a simple modification of the nanodiamonds (NDs) surface by tagging it with binding ligands or imaging probes (Wanek et al. 2023). Albumin-derived copolymer modified with desferrioxamine, is used in coating the NDs to provide a chelator for radiolabeling. The aim of the study was to evaluate both the biodistribution and the tumor accumulation of the resulting gallium-68 labeled NDs ([^68^Ga]Ga-DFO-NDs) in a xenograft model (AR42J tumor-bearing CD1 mice).

The investigators managed to successfully radiolabel the coated NDs using ^68^Ga at room temperature with radiolabeling efficiencies up to 91.8 ± 3.2% as assessed by radio-TLC. However, the in vivo experiments conclude that [[^68^Ga]Ga-DFO-NDs] is not a promising NDs imaging agent as the tumor radioactivity concentration was low compared to the higher accumulation in the liver and spleen.

The study clearly addressed most of the limitations affecting the NDs biodistribution studies, starting with the effect of tumor size and its role, the ND size (needs to be optimized), preferably below 100 nm to prolong the blood circulation time, the high-influences of both the surface charge and coating chemistry on the uptake of NPs in cells and biodistribution, finally the NDs aggregation process into the body and how fast it precipitates (within 1 h).

All listed limitations open the door wide for deep-future studies, which should concentrate on full optimizations of the surface coating as an important influence on the NDs’ biodistribution and trying to improve the blood half-life of the NDs’.

## Photocleaving radiometallated radiopharmaceuticals

*By Clemens Decristoforo*

**Fig. 2:**
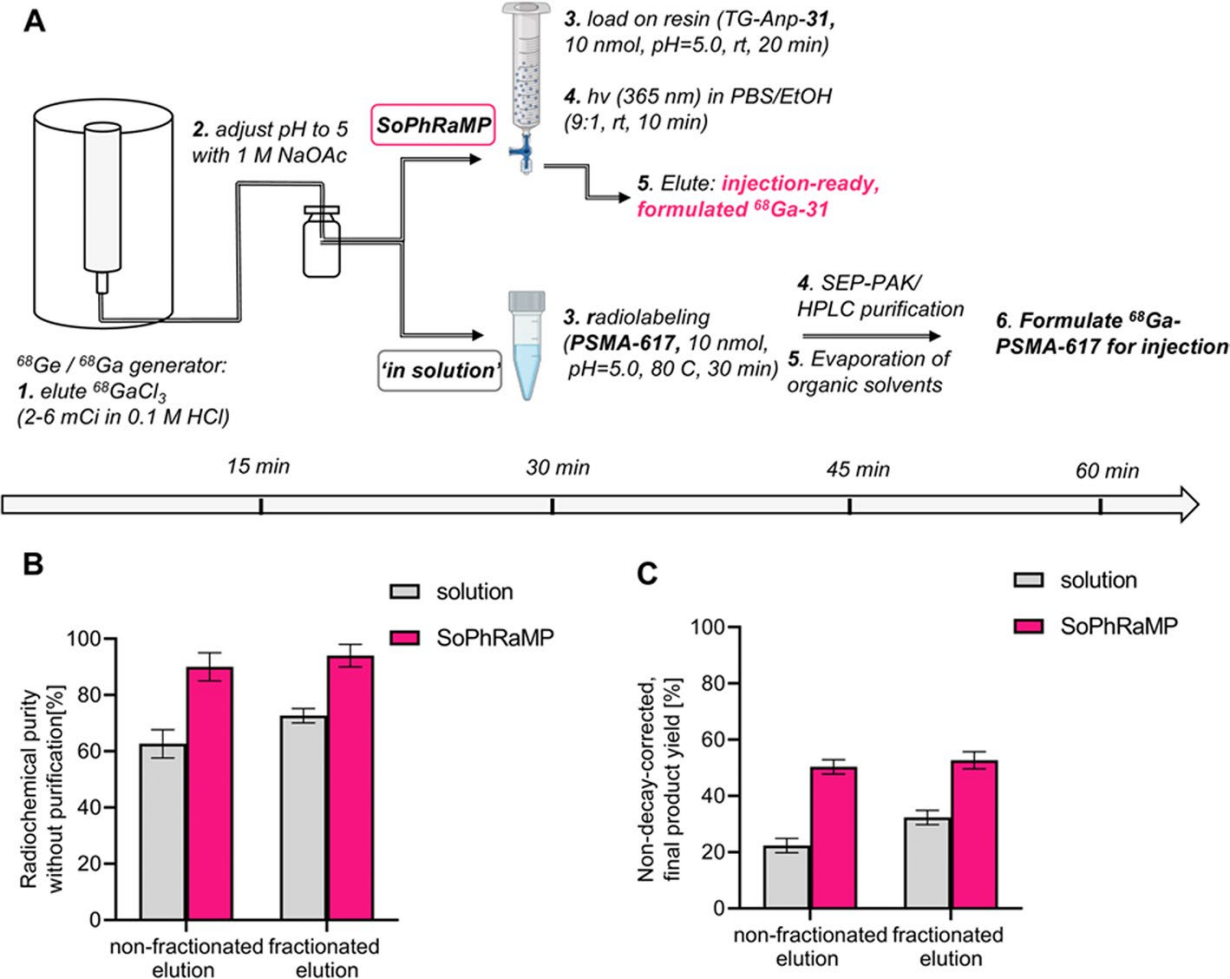
**A** Schematic description of ^68^Ga-radiolabeling procedure along with timeline for synthesis of injection-ready ^**68**^**Ga-31** using SPRP procedure and ^68^Ga-PSMA-617 using standard in solution protocol followed by secondary purification. **B** Radiochemical purity of crude labelling solution comparing solid phase vs. in-solution radiolabeling. **C** Non-decay corrected, end-of radiosynthesis yields for solid phase vs. in-solution radiolabeling. Yields reported as an average of n = 3 reactions

Even though the preparation of radiometallated, targeted radiopharmaceuticals is well established in a simple reaction of the radiometal with a buffered bifunctional ligand with almost quantitative yields, some products require extended synthesis or purification steps. In their paper (Śmiłowicz et al. 2023) the authors were inspired by recent developments of solid-phase synthesis and photochemistry. They developed an approach to combine these technologies to reach a modular radiosynthesis approach of radiometal-based radiopharmaceuticals called “Solid Phase Radiometallation and Photorelease (SPRP)”, a simple one-pot procedure based on workflows established for other food or drug applications, leading to high translatable potential. The investigators loaded different peptides with a terminal chelator via a 3-amino-3-(2-nitrophenyl)propionic acid (Anp) photocleavable moiety onto water-compatible amine functionalized resins. After addition of ^67/68^Ga or ^64^Cu in appropriate buffers to the solid phase and washing off unbound radiometal, the radiolabelled peptides were cleaved by photoirradiation at 365 nm for a few minutes, resulting in high yields, high molar radioactivity and high purity. In a proof-of-principle they could optimize the concept and prepare ^68^Ga-31, a novel PSMA ligand, on a clinical scale with excellent radiochemical purity and higher radiosynthesis yields as compared to the process for ^68^Ga-PSMA-617 in solution (Fig. 2). This interesting strategy offers new opportunities to simplify and improve the preparation of diverse radiometallated radiopharmaceuticals by immobilization on solid phase compatible with aqueous systems. To further advance this interesting concept, concrete applications have to be developed where this approach provides essential advantages over conventional preparations. Finally, regulators need to be convinced, that this is a pharmaceutically useful concept with significant advantages, the potential is there.

## Solid-phase radiometallation and photo triggered release of ready-to-inject radiopharmaceuticals

*By Hua Yang*

**Fig. 3:**
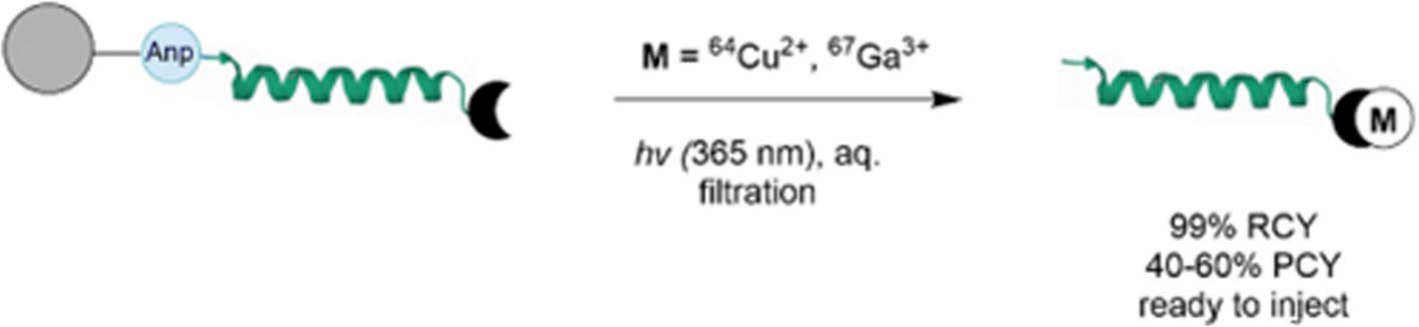
Illustration of solid-phase radiomatallation and photo triggered release. Adapted from Śmiłowicz et al. 2023

Solid-phase synthesis has long been used in peptide and oligonucleotide synthesis for the ease of purification, but is relatively underexplored in radiopharmaceutical preparation. In recent work from Boros’ group (Śmiłowicz et al. 2023), a new strategy named SPRP (solid-phase radiometal photorelease) was reported. This work combined the advantages of solid phase synthesis (no need to remove excess or unreacted metal ions) and photocatalyzed release (mild cleavage conditions and no other chemicals added). Chelator (NOTA) and short peptide sequences (BBN or PSMA) were attached to a water compatible solid support, tentagel, through a (S)-3-(Fmoc-amino)-3-(2-nitrophenyl)propionic acid (Anp) linker. After radiolabeling with ^67/68^Ga or ^64^Cu, the Anp linker can be cleaved with photo irradiation at 395 nm in aqueous solution (Fig. 3). The produced radiolabeled NOTA-peptides do not need further purification and can be injected for preclinical studies. The authors demonstrated the concept with preclinical PET imaging of ^68^Ga-NOTA-PSMA produced using this method. In comparison to the conventional liquid phase radiolabeling, this approach gives higher non-decay corrected radiochemical yield due to the short processing time. Also, the authors reported the SPRP method can tolerate Zn^2+^ and Ni^2+^ impurities better than conventional method. Future work was noted to include improving the photo cleavage sufficiency (~ 60% at 30 min) and incorporating microfluidic techniques.

## In-vivo validation of PET tracer binding site in brain by microdialysis

*By Guy Bormans*

When endogenous neurotransmitters compete for binding sites of radiotracers in brain, PET imaging may offer a means to measure variations of local (synaptic) concentrations of neurotransmitters and their modulation by pharmacological or environmental/behavioural changes. Several studies have used [^11^C]raclopride or [^18^F]fallypride to measure the synaptic concentration of dopamine (Liu et al. 2019). The affinity of the PET radioligand should be in a narrow goldilocks zone: not too high so that the tracer can be displaced by the endogenous neurotransmitter but sufficiently high to result in suitably high volume of distribution values (V_T_).

A research team from the universities of Aarhus (Denmark) and British Colombia (Canada) in cooperation with Lundbeck (Copenhagen, Denmark) have evaluated [^11^C]yohimbine as a tracer allowing quantification of synaptic noradrenaline concentration (Landau et al. 2023). Yohimbine is an antagonist of the α2-adrenergic NA receptors.

Synaptic noradrenaline concentration can be pharmacologically modulated (e.g. by amphetamine, or nisoxetine) and the resulting change in V_T_ can be correlated qualitatively with the expected change in synaptic noradrenaline. It remains however extremely difficult to quantitatively correlate the local noradrenaline concentration with the change in V_T_. In this study (Landau et al. 2023) brain in vivo microdialysis was combined with quantitative PET imaging in anesthetized Göttingen minipigs to allow quantification of local noradrenaline simultaneously with [^11^C]yohimbine PET imaging. Correct positioning of the microdialysis tip in the striatum, cortex and thalamus of the microdialysis probes was validated using CT.

The increase of noradrenaline in cortical and thalamic regions was found to correlate well with decreases in [^11^C]yohimbine PET V_T_ values, whereas no correlation was found for the striatum which is devoid of NA innervation. This well designed PET tracer validation study paves the way to application of [^11^C]yohimbine PET in future clinical CNS studies.

## Membrane-based microfluidic solvent extraction of ^68^Ga from aqueous Zn solutions: towards an automated cyclotron production loop

*By Tony Gee*

A proof-of-concept method was recently reported that allows for the microfluidic purification of batch and continuous production/purification of ^68^Ga solution targets increasing the Ga-extraction by ca. 25% compared to other extraction methods while potentially allowing for direct target recycling and re-irradiation (Trapp et al. 2023). Such technological developments raise the prospect of increased efficiency for the post target purification of liquid-based radionuclides in future.

## Are new radiopharmaceuticals making nuclear medicine still unclear?

*By Adiano Duatti*

A very interesting paper (Opalinska et al. 2023) recently reported a new advancement for imaging neuroendocrine tumours (NET) with a novel ^99m^Tc-radiolabelled somatostatin receptor (SSTR) antagonist ([^99m^Tc]Tc-TECANT1) (Novak et al. 2023). The current standard for imaging NET is the SSTR agonist [^68^Ga]Ga-DOTA-TATE/TOC. However, in low SSTR expressing tumours, the diagnostic efficacy of [^68^Ga]Ga-DOTA-TATE/TOC can be limited (Fani et al. 2017). The inherent variability of agonists’ expression was not exhaustively investigated by clinical research where immediate translation is always considered a priority. This contributes to the widespread anecdotical definition of nuclear medicine as an ‘unclear’ diagnostic tool. In contrast, radionuclide imaging is a molecularly inspired methodology. Therefore, at this very fundamental level, the development of a radiopharmaceutical always requires following a very rigorous experimental approach to achieve the highest degree of scientific certainty and reproducibility. Studies showed that [^99m^Tc]Tc-TECANT1 exhibits advantages that can outperform [^68^Ga]Ga-DOTA-TATE/TOC: *(a)* SSTR antagonists recognize more binding sites on tumour cells than SSTR agonists, thereby improving the diagnostic accuracy. *(b)* Spatial resolution of ^68^Ga-PET is poor and similar to ^99m^Tc-SPECT, but SPECT technology is approaching the sub-millimetre range, and this could greatly enhance the diagnostic sensitivity and precision. In short, [^99m^Tc]Tc-TECANT1 can potentially contribute to make nuclear medicine less ‘unclear’.

Unfortunately, a recent commentary (Ah-Thiane et al. 2023) with the rather discomforting title ‘PSMA Is Not Specific to Prostate Cancer’, leads to the opposite conclusion. After thousands of papers praising the potential of PSMA-based radiopharmaceuticals for the precision medicine of prostate cancer, including the current emphasis on the elusive nature of ^225^Ac-compounds (Stein and Kerlin 2019; Wilson 2019), this rings an alarm bell that the ‘unclear medicine’ narrative is not going to disappear completely.

## Overcoming limitations of conventional radiolabeling, MMAAC enables preparation of radiopharmaceuticals with high molar activity

*By Xing Yang*

PET is a molecular imaging technique that allows quantitative visualization of biological processes in vivo. PET tracers with high molar activity are essential for minimizing biological target occupancy and facilitating the detection of low-target-expressing tumors. However, conventional radiolabeling is performed in the presence of a large excess of ligand, resulting in relatively low molar activity. A recent publication (Śmiłowicz et al. 2023) describes an innovative method called metal-mediated, autolytic amide bond cleavage (MMAAC), which utilizes metal complexation of Lewis acids (e.g. Ga^3+^ and Sc^3+^) as a trigger to release (radio-)metallopharmaceuticals, offering a potential solution to this dilemma.

**Fig. 4:**
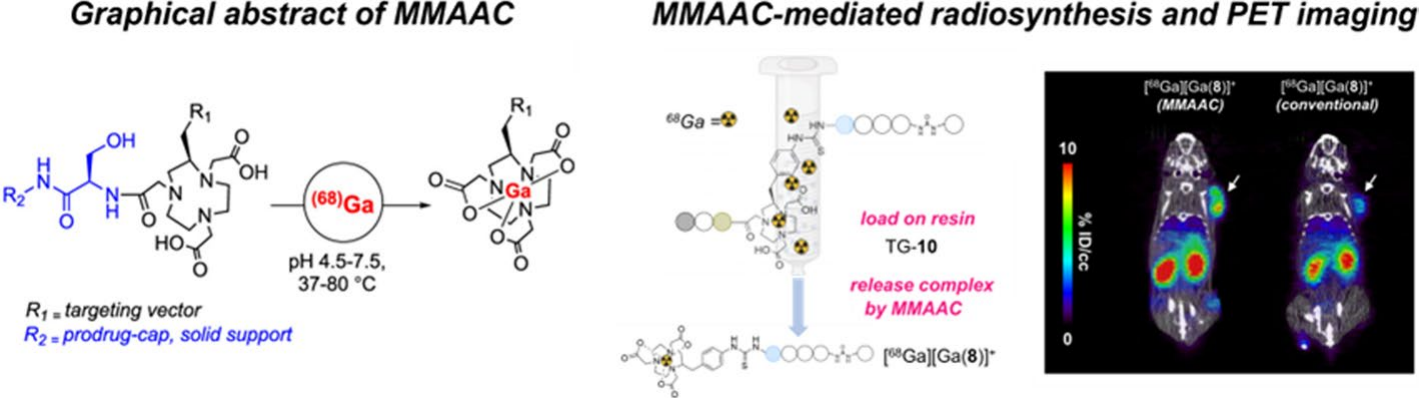
Graphical abstract image and proof-of-concept of MMAAC. (Adapted with permission from Śmiłowicz et al. Copyright 2023 American Chemical Society.)

The authors optimized the kinetics and verified the mechanism of the MMAAC reaction. Then, the authors designed and synthesized TG-**10**, which contains a cleavable serine-glycine capping group to trigger the selective cleavage of MMAAC and a PSMA-targeting peptide sequence for tumor targeting, as a proof-of-concept study. ^68^Ga was directly eluted from a generator and reacted with TG-**10** on solid support. After radiolabeling, MMAAC released only the ^68^Ga-labeled [^68^Ga]Ga-**8** into solution, while the unlabeled precursor remained on the solid support. PET imaging and biodistribution experiments in mice bearing low PSMA-expressing tumors demonstrated significantly higher tumor uptake of this high molar activity radiotracer compared to conventional solution-phase radiolabeling products (Fig. 4).

This work highlights the innovative potential of MMAAC in preparing high molar activity radiopharmaceuticals. Future outlooks include expanding beyond specific metal ions such as ^177^Lu for therapeutic purpose, and exploring the generalizability of this method to enhance radiopharmaceutical molar activity.

## [^99m^Tc]Tc-ADAPT6 and [^99m^Tc]Tc-(HE)_3_-G3 are promising probes for imaging of HER2 expression in breast cancer.

*By Junbo Zhang*

Human epidermal growth factor receptor 2 (HER2) is overexpressed in some breast cancers. Imaging the expression of HER2 in breast cancer may predict response to HER2-targeted treatments. Because nuclear molecular imaging, such as PET imaging and single-photon emission computed tomography (SPECT) imaging are noninvasive and can afford information about HER2 expression, it is of great value to develop effective radiolabeled probes for imaging of HER2 expression.

Technetium-99 m has been the most widely used radionuclide for SPECT. Thus, to develop easily available ^99m^Tc-labelled probes for imaging of HER2 expression is of great importance. ADAPT6 and DARPin (designed ankyrin repeat proteins) G3, as scaffold proteins with high affinity to HER2, can be radiolabeled with ^99m^Tc to form [^99m^Tc]Tc-ADAPT6 and [^99m^Tc]Tc-(HE)_3_-G3 for imaging of HER2 expression, respectively. Recently, a comparison of [^99m^Tc]Tc-ADAPT6 and [^99m^Tc]Tc-(HE)_3_-G3 for imaging of expression of HER2 in breast cancer has been reported (Bragina et al. 2023). The authors performed the study in 11 patients with HER2-positive tumors. Both of the two tracers can detect HER2-positive breast cancer. The uptake of [^99m^Tc]Tc-(HE)_3_-G3 in primary tumors was significantly lower than that of [^99m^Tc]Tc-ADAPT6. [^99m^Tc]Tc-ADAPT6 exhibited a higher uptake in soft tissue lesions and less uptake in the liver, suggesting its potential to be a better tracer for imaging of HER2-positive cancer. Further studies of [^99m^Tc]Tc-ADAPT6 for imaging of HER2 expression in breast cancer are expected.

## Synthesis and preclinical evaluation of 2‑(4‑[^18^F]Fluorophenyl)imidazo[1,2-h][1,7]naphthyridine ([^18^F]FPND-4): an Aza-fused tricyclic derivative as positron emission tomography tracer for neurofibrillary tangle imaging

*By Hongmei Jia*

The stages of tau pathology appear to correlate well with the progression of cognitive impairment. An optimal PET radiotracer for in vivo tau imaging will serve as a powerful tool to investigate the time course of tau accumulation in AD patients. However, for the first-/second-generation tau radiotracers including [^18^F]flortaucipir, [^18^F]MK6240, [^18^F]PI2620 and [^18^F]APN-1607, there are still some limitations including slight metabolic defluorination, off-target binding to choroid plexus, monoamine oxidase A/B (MAO-A/B), and other targets.

**Fig. 5:**
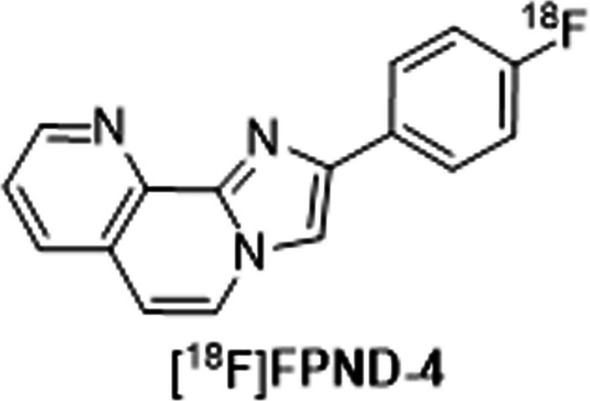
Chemical structure of the Tau tracer [^18^F]FPND-4

Recently, researchers from Beijing Normal University (Liu et al. 2023a) developed [^18^F]FPND-4, a novel aza-fused tricyclic derivative, as a PET tracer for neurofibrillary tangle imaging (Fig. 5). The new imidazole naphthyridine scaffold was discovered and evaluated by the same group in their previous work (Liu et al. 2023b). The absence of off-target binding of FPND-4 to Aβ and MAO-A/B was validated. Notably, this compound possessed an outstanding affinity to 3R/4R mixed tau tangles on AD brain slices (IC_50_ = 2.8 nM). In ex vivo biodistribution in mice, [^18^F]FPND-4 presented desirable initial brain uptake (3.95 ± 0.37% ID/g at 2 min) and rapid clearance (brain_2 min/60 min_ = 33). In vitro autoradiography studies demonstrated intense autoradiographic signals of [^18^F]FPND-4 on the hippocampus and temporal lobe of all AD and PART brain slices, loaded with different kinds of tau deposits. In dynamic PET imaging in rodents and rhesus monkey, [^18^F]FPND-4 exhibited high initial brain uptake and rapid washout among all brain regions devoid of tau pathology. This tracer also displayed minimal defluorination and few abnormal radioactive accumulations in non-target regions. Taken together, [^18^F]FPND-4 fulfills the requirements for an tau-specific imaging agent and warrants further investigation in humans.

## Sustainable alternatives for the production of ^99m^Tc, an outstanding debt?

*By Marcela Zubillaga*

Despite the sustained growth of new radioisotopes and radiopharmaceuticals, ^99m^Tc continues to be one of the most widely used radioisotopes in diagnostic nuclear medicine.

During the first decade of the 2000s, shutdowns of ^99^Mo-production reactors demonstrated the fragility and unpredictability of the global medical isotope supply system (Knight 2009). The global interruptions of ^99^Mo-supply, aging reactors, and the exorbitant costs of their maintenance have accelerated the search for alternative sources of ^99m^Tc (Guerin et al. 2010). Recently, the COVID-19 pandemic revealed other weaknesses in the ^99^Mo-production and distribution chain for the local/regional production of ^99^Mo/^99m^Tc-generators.

In most of the published works, the alternative consists in the production of ^99m^Tc in cyclotrons for medical use by bombarding different targets with protons. This alternative approach is valid as long as the production of this radioisotope alternates with PET radioisotopes. In situtations where this is not the case, a proposed new strategy (Johnstone et al. 2023) allows for the simultaneous production of ^18^F and ^99m^Tc in a low energy biomedical cyclotron available at many national facilities.

This study demonstrates that it is feasible to co-produce ^99m^Tc in parallel with ^18^F using a hybridized system based upon liberated neutrons from the ^18^O(*p*,*n*)^18^F with a low energy biomedical cyclotron. The goal of this study is to demonstrate the possible the co-production of both isotopes defining for its scaling up: Mo-sample design and placement, target mass/volume, irradiation time, and neutron flux.

More important things to be considered are related to the system platform for the appropriate isolation of ^99m^Tc from Mo-targets and the national GMP and radioprotection regulations for its safety production.

## Catering for an unusual diagnostic need

*By Sietske Rubow*

In radiochemistry and radiopharmacy, there is always a search for novel products and original compounds or faster, better production methods. Published work largely focuses on exciting new discoveries. Sometimes, however, it may be necessary to revisit Nuclear Medicine methods and radiopharmaceuticals that were developed many years ago and are infrequently used, considering how they can be adapted to our current technology (e.g. total body PET) and refreshed to continue providing the best possible service for our patients.

One of the earliest reports on use of heat-damaged radiolabelled red blood cells for splenic imaging was published more than 60 years ago (Winkelman et al. 1960). Since then, this technique has been used, especially SPECT imaging with ^99m^Tc-labelled heat-damaged erythrocytes, where the distinction between splenic and other adjacent tissue is difficult. The group of Drescher have added a new chapter to the story of this perhaps infrequently applied but very useful technique (Drescher et al. 2023). To overcome the spatial resolution limitations associated with SPECT, they employed [^68^Ga]Ga-oxinen (Thompson et al. 2018) to prepare heat-damaged erythrocytes. While an automated synthesis routinely prepared the [^68^Ga]Ga-oxine, the erythrocytes are isolated. To shorten the total preparation time, the denaturation and radiolabelling of the red blood cells were combined in a single heating step. The protocol was established to meet Good Radiopharmacy Practice requirements and quality control followed relevant established procedures for red cell labelling and standards of the European Pharmacopoeia.

It is encouraging to see that time and effort is spent to provide an updated version of a less frequently used diagnostic procedure, while ensuring that the radiopharmaceutical meets the requirements for patient administration.

## In vivo metabolic imaging of [1-^13^C]Pyruvate-d3 hyperpolarized by reversible exchange with parahydrogen

*By Peter Caravan*

Hyperpolarized ^13^C MRI with pyruvate enables direct assessment of metabolic flux by quantitative imaging of pyruvate and its metabolites alanine and lactate. A hyperpolarized molecular MRI scan takes about one minute and employs non-ionizing radiation. Clinical production of [1-^13^C]Pyruvate involves dynamic nuclear polarization for an hour in a superconducting magnet at very low temperature followed by rapid unfreezing and sample dissolution. An alternative approach called SABRE is to use parahydrogen in the presence of an iridium catalyst to hyperpolarize pyruvate. SABRE polarization is fast, performed at room temperature in a small, low-cost device and can be repeated. However, SABRE requires organic solvents to achieve high polarization and an iridium catalyst, both of which must be removed to provide a biocompatible formulation. After hyperpolarization the ^13^C will begin to relax and so there is very little time available for solvent exchange. De Maissin (Maissin et al. 2023) used an improved SABRE polarization process using spin-lock induced crossing and combined this with very rapid solvent evaporation and catalyst filtration steps to achieve up to 16% ^13^C polarization of [1-^13^C]Pyruvate with methanol and Ir levels below toxic levels (de Massin et al. 2023). These innovations enabled the first preclinical imaging studies with SABRE hyperpolarized pyruvate and brings this efficient and cheap process (compared to DNP) closer to clinical applications.

## Opportunities and potential challenges of using terbium-161 for targeted radionuclide therapy in clinics

*By Jan Rijn Zeevaart*

The field of nuclear medicine is fast paced, has always been, and seems to accelerate. This innovative aspect (to have new radioisotopes) is both its biggest asset but also its potential downfall. The pharmaceutical industry is not used to a completely new approach every 5 years. In our quest for the magic bullet it has always been postulated that the isotope of choice will transition from beta to alpha to Auger, the pace of this transition has surprised all as ^161^ Tb is the first Auger emitter (although a combination with betas) that is now becoming widely discussed. A recent review takes stock of ^161^ Tb; where it is in the drug development pipeline and the potential challenges it will face reaching the clinic (Müller et al. 2023).

The use of ^161^ Tb is not new, in 1995 an evaluation of ^161^ Tb-labelled DTPA octreotide was published (de Jong et al. 1995). However, the credit for taking ^161^ Tb from concept to a real possibility in Nuclear Medicine should go to the PSI/ETH group. Terbium-161 has similar decay properties to ^177^Lu. It is postulated, however, that the co-emission of conversion and Auger electrons make ^161^ Tb superior. These short-ranged electrons may effectively eliminate microscopic metastases that are not even visible on a PET image, but responsible for relapse and metastatic spread (Bernhardt et al. 2021).

Proving this postulate is what Mueller and co-workers set out to do since 2011. [^161^ Tb]Tb-PSMA-617 reduced the viability and survival of PSMA-expressing PC-3 PIP tumor cells more than twice as effectively as [^177^Lu]Lu-PSMA-617 (Mueller et al. 2019). It was experimentally demonstrated that [^161^Tb]Tb-DOTA-LM3 was many-fold more effective in reducing the viability of AR42J tumor cells than its ^177^Lu—labelled counterpart—despite the fact that more than 90% of this somatostatin receptor antagonist are localized at the cellular membrane (Borgna et al. 2022). The authors then describe the likelihood of widespread clinical translation of terbium-161 will be feasible and realistic in the near future under the following aspects: production and availability, Measurability, Radioligand preparation are all favourable for ^161^Tb.

For pharmacokinetics ^161^Tb, unlike other theranostic pairs where the switch from one radiometal to another can alter the tissue distribution, this is not expected to occur when using ^161^Tb instead of ^177^Lu as proven in preclinical (Muller et al. 2019; Borgna et al. 2022) and first-in-human studies (Baum et al. 2021).

From the above, the future for ^161^Tb indeed looks bright and therefore no wonder that several clinical trials have been initiated, or are currently ongoing, in Switzerland, Australia, (VIOLET trial) and Germany (REALITY trial). However, one has to concur with the authors that it will likely take years to demonstrate the expected superiority of ^161^Tb over ^177^Lu in eliminating microscopic tumors.

## Production of ^67^Cu in compact cyclotrons: towards the spread use of true theranostic pairs with copper radioisotopes

*By Miguel A. Avila-Rodriguez*

Over the past decade theranostics have revolutionized nuclear medicine applications bringing new hope for patients suffering several types of cancer. The combination of using a vector molecule labeled with suitable radionuclides to first identify and then treat primary and metastatic tumors have opened the door to a new paradigm for medical care, moving nuclear medicine practices towards a personalized treatment model. The most widely used theranostic matched pair of radionuclides is by far ^68^Ga/^177^Lu, and in practice it is assumed that the biological behavior of the molecular vector labeled with both radionuclides is identical, which is not necessarily true. That is why the idea of using true theranostic radionuclide pairs have aroused interest in recent years, and in this regard copper radioisotopes are excellent candidates.

A recent publication of the Helmholtz-Zentrum Dresden-Rossendorf group reports on their experience producing ^67^Cu, a promising beta-emitter for targeted radionuclide therapy, via the ^70^Zn(p,α)^67^Cu reaction (Brühlmann et al. 2023). Thick depositions (> 100 mg/cm^2^) of isotopically enriched ^70^Zn (> 97%) were efficiently electroplated on gold and silver substrates and irradiated with currents over 50 µA of 17 MeV protons, for up to 12 h, obtaining ^67^Cu with high radionuclide purity (> 99.5%) and apparent molar activity (80 MBq/nmol), in a yield close to the GBq range, and real possibilities of improvement. They also efficiently recovered the enriched ^70^Zn for subsequent electrodepositions, reducing the production cost. The proven feasibility of producing ^67^Cu in compact cyclotrons broadly available worldwide, in addition to their diagnostic pairs ^61^Cu/^64^Cu, open new opportunities for expanding theranostics applications using true pairs.

## Are we close to having a PET radiotracer form imaging alpha synuclein in Parkinson’s disease?

*By Robert H. Mach*

Alpha synuclein (αsyn) is a 140 amino acid intrinsically disordered protein that has a high expression in neurons. Under normal conditions it is a soluble protein that is thought to aid in the docking of synaptic vesicles to release zones in synaptic terminals. Through a mechanism that is poorly understood, αsyn can fold into a beta pleated sheet and form insoluble protein aggregates ranging from oligomers to protofibrils to fibrils. The formation of insoluble protein aggregates of αsyn fibrils represents the hallmark feature of a family of neurodegenerative disorders referred to as the “synucleinopathies” with the most prominent being Parkinson’s disease (PD). There are multiple strategies under development to reduce αsyn aggregates in the CNS as a means of delaying disease progression in PD. The availability of a PET radiotracer that can image αsyn aggregates in PD brain would represent an important tool for measuring the efficacy of these therapeutic strategies. Unfortunately, the development of a PET radiotracer for this purpose has been very challenging.

**Fig. 6:**
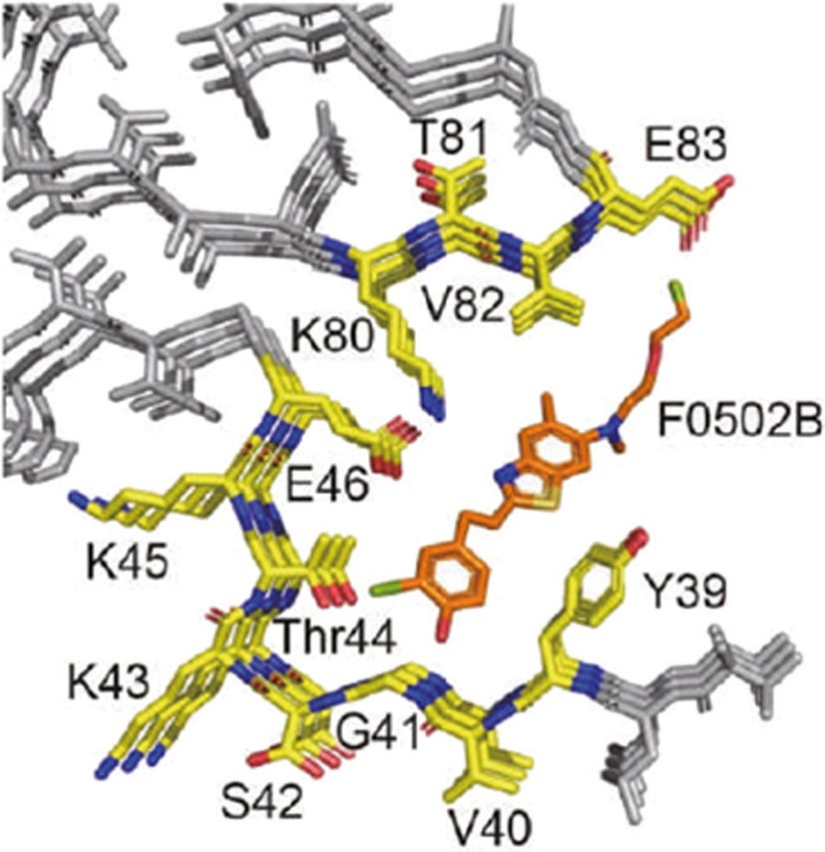
Interaction of F0502B with α-synuclein. With permission Creative Commons users license 561837064078

A recent radiotracer that has shown promise in postmortem studies of PD brain is the benzothiazole analog, [^18^F]F0502B (Xiang et al. 2023). Although this compound is structurally-similar to PiB (Aβ radiotracer) and PM-PBB3 (mixed tau/αsyn radiotracer), evidence reported by this group suggests remarkable specificity for αsyn over Aβ and tau. An important milestone in the characterization of this radiotracer was the resolution of a cryoEM structure of F0502B bound to recombinant αsyn fibrils. Although the brain uptake was somewhat low in PET imaging studies in nonhuman primates, it is quite likely that this limitation can be overcome with further analog development. A potential concern is the key π-π stacking interaction between the TYR-39 and the phenol ring of F0502B (Fig. 6). The phosphorylation of TYR-39 represents a key post-translation modification found in PD brain, which could interfere with the binding of [^18^F]F0502B to αsyn in vivo. Regardless of this minor point, the identification of a radiotracer that is capable of imaging αsyn in PD would represent a major advance to the field of PET, and this group appears to be well on its way to meeting this challenge.

## Costs of in-house preparation and treatment with [^177^Lu]Lu-PSMA I&T

*By Jeroen Hendrikx*

The EANM position paper on the in-house preparation of radiopharmaceuticals states that major clinical breakthroughs in Nuclear Medicine were based on the use of in-house preparations (Hendrikse et al. 2022). In-house preparations definitely helped in the fast acceptance of ^177^Lu-labeled PSMA-targeted radioligand-therapy prior to registration of Pluvicto^®^. However, for balanced access to new therapies in routine clinical care, we also have to focus on value-based healthcare to get reimbursement of costs. Therefore, reports of economic evaluations are essential. Quist et al. compared treatment costs of in-house produced [^177^Lu]Lu-PSMA-I&T and compared it to costs of radium-223 therapy (Quist et al. 2023). Based on an economic evaluation model, it was concluded that treatment costs per patient with the in-house preparation of [^177^Lu]Lu-PSMA-I&T (VISION trial scheme) were higher than radium-223 therapy (€47.456 vs € 30.905, respectively). Although this analysis is great for transparency of treatment costs, a major draw-back in this comparison is not including treatment outcome in the analysis, making it not possible to come to a cost-effectiveness comparison between therapies. Assuming comparable clinical outcome of [^177^Lu]Lu-PSMA-617 and [^177^Lu]Lu-PSMA-I&T (Kratochwil et al. 2023), cost-effectiveness of the in-house preparation of [^177^Lu]Lu-PSMA-I&T can be estimated. For [^177^Lu]Lu-PSMA-617, a gain of 0.42 quality-adjusted life year (QALY) is reported (Mehrens et al. 2023). Therefore, the estimated cost-effectiveness of the in-house preparation of [^177^Lu]Lu-PSMA-I&T is €47.456/0.42 = €112.990 per QALY. Although this is just a rough estimation, knowledge of the cost-effectiveness of our in-house produced radiopharmaceuticals will help in fast and broad acceptance of new developments.

## New steps in nano-radiopharmaceutical approach

*By Tamer Sakr*

Nanocarriers have started to play an efficient role for enhancing drug delivery and therapeutic efficacy (Faheela et al. 2022). Many studies have evaluated the advances in use of nanocarriers for cancer treatment based on enhanced permeability and retention (EPR) phenomena (Xiao et al. 2021). EPR allows proper drug delivery to cancer tissues that enhance drug pharmacokinetics, and precise targeting that leads to fewer side effects and drug resistance (Xiao et al. 2021). Other new approaches evaluated the use of nanocarriers in radioisotope delivery for cancer diagnosis and therapy (Jalilian et al. 2022). A new publication discussed the optimization of radioiodinated acemetacin-loaded niosomes for cancer therapy (Shewaiter et al 2022). The team used a D-optimal design to investigate the effects of independent variables such as cholesterol amount, span 60 amount, and hydration time on the dependent variables. The particle size of the niosomes was around 200 nm. The stability study, particle size, zeta potential, and entrapment efficiency remained stable throughout the study. The in vivo pharmacokinetic differences in mice models, involving evaluation of C_max_, T_max_, T_1/2_, MRT, AUC_0–24_, tumor AUC_0–24_, and the tumor drug targeting efficiency (AUC target/AUC non-target), indicated higher drug accumulation in the tumor compared to the blood and other body organs (Shewaiter et al. 2022). Overall, the report showed that niosomal formula increased tumor uptake of radioiodinated acemetacin by passive targeting of the nanosized niosomes with a possibility of combining the chemotherapeutic effect of acemetacin and radiotherapeutic effect of ^131^I in one therapeutic agent.

## Cyclotron production of ^43^Sc and ^44g^Sc from enriched [^42^Ca]CaO, [^43^Ca]CaO, and [^44^Ca]CaO targets

*By Giuseppe Carlucci*

The highlighted article explores the production of the radioisotopes ^43^Sc and ^44g^Sc using cyclotron irradiation of enriched calcium oxide (CaO) targets: ^42^Ca(d,n)^43^Sc, ^43^Ca(p,n)^43^Sc, ^43^Ca(d,n)^44g^Sc, ^44^Ca(p,n)^44g^Sc, and ^44^Ca(p,2n)^43^Sc (Becker et al. 2023). The article delves into the methods and techniques employed in producing these isotopes. Both ^43^Sc and ^44g^Sc emit positrons with lower mean energies than ^68^Ga, have high positron branching ratios, and are chemically identical to the therapeutic radionuclide ^47^Sc. Additionally, the ionic radius and coordination behavior of scandium are better matched to lutetium than gallium.

The study covers target preparation, irradiation conditions, radiochemical separation, yield measurements, radionuclidic purity, and PET image quality. The results of this work demonstrate that proton and deuteron bombardment of isotopically enriched CaO targets produce high yield and high radionuclidic purity ^43^Sc and ^44g^Sc (Table 1).

**Table 1:** Observed production yields for the 5 routes investigated

Reaction	Isotopic enrichment (%)	Incident energy (MeV)	Target mass (mg)	Target thickness (MeV)	Production yield (mCi/μAh)	Yield corrected for beam intercept (mCi/μAh)	Predicted yield (mCi/μAh)	Radionuclidic purity (%)
^42^Ca(d,n)^43^Sc	93.58 ± 0.05	5.8	62 ± 4	5.8	0.82 ± 0.05	1.49 ± 0.09	1.66*	99.36 ± 0.05
^42^Ca(p,n)^43^Sc	83.93 ± 0.10	13.6	148 ± 1	4.6	6.19 ± 0.81	12.64 ± 0.92	15.41*	87.81 ± 0.03
^42^Ca(d,n)^44^Sc	83.93 ± 0.10	5.8	61 ± 2	4.6	6.19 ± 0.81	12.64 ± 0.92	15.41*	87.81 ± 0.03
^42^Ca(p,n)^44^Sc	98.89 ± 0.02	13.6	150 ± 2	4.7	11.71	23.92	36.83*	99.71 ± 0.05
^42^Ca(p,2n)^43^Sc	98.89 ± 0.02	15.2	151 ± 2	4.3	0	0	0	–
^42^Ca(d,n)^44^Sc	98.89 ± 0.02	15.2	151 ± 2	4.3	10.92 ± 1.32	22.6. ± 0.57	34.30*	99.51 ± 0.06

With permission from Front Chem 2023.1167783 doi 10.3389 (Creative Commons Attribution Licence CC BY)

The molar activity of ^43/44g^Sc produced through a 30–60 min, 13.6 MeV proton energy and 5 μA current irradiation of either [^43^Ca]CaO or [^44^Ca]CaO targets was 48 ± 11 GBq/μmol (1.3 ± 0.3 Ci/μmol) (n = 3) at the end of chemistry. Additionally, 37 ± 5 MBq (1 ± 0.14 mCi) aliquots of ^43/44g^Sc were labeled with DOTA-TATE at a molar activity of 7.14 MBq/nmol (0.2 mCi/nmol) and assayed using HPLC. Radio-HPLC chromatograms show near quantitative labeling (> 98%). Free ^43/44g^Sc was eluted with the solvent front, and the final product ^43/44g^Sc-DOTA-TATE was eluted at 15.6 min. The contrast, C_rod_, was compared between ^43^Sc, ^44g^Sc, ^43/44g^Sc, ^18^F, ^64^Cu, ^68^Ga radionuclides on two PET/CT scanners. Radioisotopes of scandium demonstrated image quality between the low and high positron range radionuclides where the smallest diameter rods are indistinguishable, but resolution improves with the 4 mm diameter rods.

Overall, this research contributes to advancing nuclear medicine by addressing the technical aspects of cyclotron-produced ^43^Sc and ^44g^Sc. As the field of radiopharmaceuticals continues to evolve, such studies are critical for expanding the repertoire of available diagnostic tools and therapeutic options, ultimately benefiting patient care.

## Targeting the stimulator of interferon genes (STING) protein with a novel ^18^F-labeled acridone derivative

*By Jean N. DaSilva*

STING is a transmembrane protein which plays important roles in cancer, cytotoxic therapies, and various infections. Several immunotherapy approaches designed to initiate anti-tumor immune response were shown to activate the cGAS-STING pathway and the therapeutic effects of the treatments are correlated with the STING expression within the tumor microenvironment (Corrales and Gajewski 2015). STING is thus a promising immunotherapeutic target for cancer treatment. Interestingly, intratumoral injection of various STING agonists produced therapeutic effects in multiple mouse tumor models and several STING agonists are currently tested in preclinical and clinical trials. Development of selective PET imaging probes would permit to visualize STING expression. Liu and colleagues recently reported the synthesis and evaluation of their most recent ^18^F-labeled analogue of the STING agonist acridone, [^18^F]F-CRI1 (Liu et al. 2023c, d).

[^18^F]F-CRI1 prepared via the copper-catalyzed azide–alkyne cycloaddition click reaction displayed nanomolar binding affinity for STING, and high stability in saline and mouse serum. Small animal PET studies in CT26 tumor-bearing mice revealed gradual accumulation of [^18^F]F-CRI1 in the tumor reaching a peak at 1 h post-injection. Significant reduction of the tracer uptake both in vitro in cells and in the tumor by microPET with the STING agonist DMXAA demonstrates binding specificity. High tumor-contrast images and binding specificity suggest that [^18^F]F-CRI1 has potential for imaging STING. However, high accumulation in the gastrointestinal tract and gallbladder suggests that additional structural modifications on the probe may improve its potential to study STING in the tumor and potentially guide STING-related treatments with PET imaging.

## Conclusions

The latest trends in radiopharmacy and chemistry are highlighted.

## Data Availability

Datasets mentioned in this article can be found in the cited articles.

## Abbreviations

AI: Artificial intelligence
Αsyn: α-Synuclein
C_rod_: Comparison of contrast
DOTA: 2,2′,2″,2″-(1,4,7,10-Tetraazacyclododecane-1,4,7,10-tetrayl)tetraacetic acid
EPR: Enhanced permeability and retention
MMAAC: Metal-mediated autolytic amide bond cleavage
ND: Nanodiamond
NET: Neuroendocrine tumor
PET: Positron emission tomography
PRRT: Peptide receptor radionuclide therapy
PSMA: Prostate specific membrane antigen
SPECT: Single photon emission computed tomography
SSTR: Somatostatin receptor
